# Food Safety through Natural Antimicrobials

**DOI:** 10.3390/antibiotics8040208

**Published:** 2019-10-31

**Authors:** Emiliano J. Quinto, Irma Caro, Luz H. Villalobos-Delgado, Javier Mateo, Beatriz De-Mateo-Silleras, María P. Redondo-Del-Río

**Affiliations:** 1 Department of Nutrition and Food Science, Faculty of Medicine, University of Valladolid, 47005 Valladolid, Spain; irma.caro@uva.es (I.C.); bdemateo@yahoo.com (B.D.-M.-S.); pazr@ped.uva.es (M.P.R.-D.-R.); 2 Institute of Agroindustry, Technological University of the Mixteca, Huajuapan de León, Oaxaca 69000, Mexico; vidluz@mixteco.utm.mx; 3 Department of Hygiene and Food Technology, Faculty of Veterinary Medicine, University of León, 24071 León, Spain; jmato@unileon.es

**Keywords:** natural antimicrobials, preservation, plants, spices, bacteria, viruses, algae, mushrooms, bacteriocins, bacteriophages

## Abstract

Microbial pathogens are the cause of many foodborne diseases after the ingestion of contaminated food. Several preservation methods have been developed to assure microbial food safety, as well as nutritional values and sensory characteristics of food. However, the demand for natural antimicrobial agents is increasing due to consumers’ concern on health issues. Moreover, the use of antibiotics is leading to multidrug resistant microorganisms reinforcing the focus of researchers and the food industry on natural antimicrobials. Natural antimicrobial compounds from plants, animals, bacteria, viruses, algae and mushrooms are covered. Finally, new perspectives from researchers in the field and the interest of the food industry in innovations are reviewed. These new approaches should be useful for controlling foodborne bacterial pathogens; furthermore, the shelf-life of food would be extended.

## 1. Introduction

**Figure 1:**
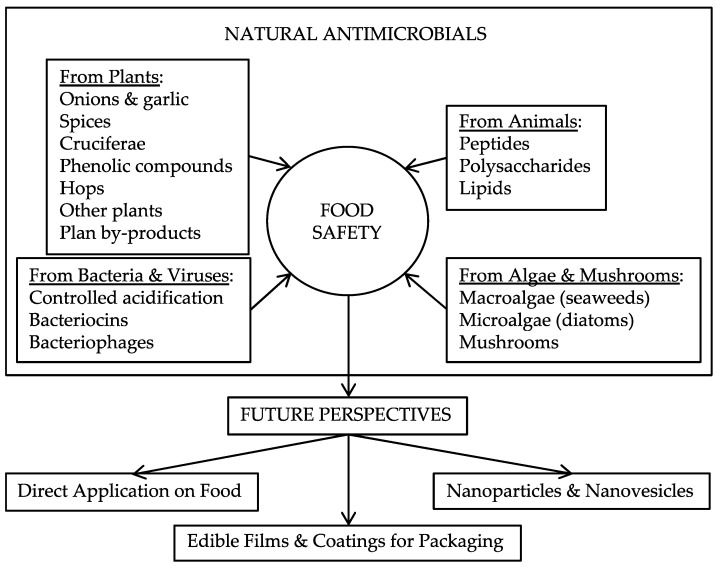
Overview of natural antimicrobials and their role in food safety.

Microbial pathogens are the cause of many foodborne diseases after the ingestion of contaminated food. Several preservation methods have been developed to assure microbial food safety, as well as nutritional values and sensory characteristics of food. Those methods sometimes have undesired effects on the nutritional and/or organoleptic aspects of food; synthetic preservatives are well known for causing health problems such as allergic reactions: nitrates, benzoates, sulfites, sorbates, formaldehyde, and phenolic antioxidants are good examples [1,2]. The use of natural antimicrobial food preservatives—biopreservation—could ensure the safety and quality of food being an alternative to other systems of preservation such as chemical or thermal ones. An excellent overview of natural antimicrobials applications can be seen in [1]. Biopreservation uses natural preservatives against a high number of pathogenic microorganisms related to food; those preservatives are obtained from animals, plants, bacteria, as well as mushrooms, algae, and viruses [2]. Figure 1 shows a general view of natural antimicrobials and their different roles in food safety.

The demand for natural antimicrobial agents is expected to increase steadily for replacing synthetic compounds [3]. A novel trend is arising from health-conscious consumers expecting that natural antimicrobials act only against foodborne pathogens leaving the consumers’ microbiome out of their scope [1]. The negative effect of some synthetic preservatives on consumers’ health is leading to more research to evaluate that natural antimicrobials fulfil food safety regulations [4]; the inadequate use of antibiotics leading to multidrug-resistant microorganisms also justify and reinforce the focus on natural antimicrobials [2]. Natural antimicrobials ensure food safety from a new perspective increasing its shelf-life; furthermore, their direct incorporation to different foods from different origins such as meat or vegetables as well as to their packaging give, as a result, the extension of their shelf-life [1,5]. They also constitute a viable alternative to microbial resistance caused by antibiotics.

Recent studies comparing natural derivatives from plants with synthetic antimicrobials have shown that natural substances could be safer [3,6,7]. The mechanisms of action of natural antimicrobials include the rupture of the cell membrane, affect the nucleic acids mechanisms, the decay of the proton motive force, and depletion of adenosine triphosphate (ATP). Antimicrobials from plants (polyphenols, essential oils), animals (lysozyme, lactoperoxidase, lactoferrin), metabolites from microorganisms, or extracts from algae use those mechanisms of action against foodborne bacteria [1,8].

## 2. Natural Antimicrobials from Plants

Herbs and spices have most of the antimicrobials derived from plants [9,10,11]. These compounds have different structural configurations, having different antimicrobial actions against foodborne pathogens [12]. A fine review showing the different structural variations of plant-derived components and their effect on their antimicrobial capacities was published by Gyawali and Ibrahim [2]. The structural configuration of these compounds has big impact on their antimicrobial action, i.e. the hydroxyl (−OH) groups are thought to be the cause; the reason behind that fact is the interaction of the hydroxyl groups with the bacterial cell membrane disrupting its structures and causing leakage of its components.

Growing interest in using antimicrobial plant-derived extracts is caused by the need to reduce the use of synthetic additives in food [13]. Antioxidant capacity usually joins the antimicrobial characteristics of these natural products; both properties together in one molecule makes the compound even more effective [1]. Plants and herbs (oregano, garlic, parsley, sage, coriander, rosemary, and lemongrass), spices (cinnamon, clove), oils (citral) or organic compounds (vanillin) have been used alone for their antimicrobial and antioxidant properties or in combination with other techniques for food preservation [14,15,16]. These authors also reported lower activity from products such as ginger, pepper, cumin, chilli, and curry. Gutierrez et al. [14] assessed combinations of essential oils from thyme, sage, rosemary, oregano, lemon, and basil against different microorganisms: *Bacillus cereus*, *Escherichia coli*, *Listeria monocytogenes* and *Pseudomonas aeruginosa*. Oregano showed efficacy against *B. cereus*; furthermore, the oregano combinations with basil or thyme were active against *B. cereus*, *E. coli* and *P. aeruginosa*. These authors also studied the effect of the pH and different ingredients from foods on the activity of thyme and oregano against *L. monocytogenes*; the kinetic parameters of the microorganism were more affected in foods with acidic pH and a high content in proteins. Proestos et al. [15] studied extracts from five plants—meadowsweet, hawthorn, polygonum, silverweed, and little robin—showing their antioxidant capacity, with total phenolic contents between 7.2–28.2 gallic acid equivalents/mg or mL; their activity against the microorganisms showed that Gram-negative bacteria were less sensitive than Gram-positive. Numerous studies have been carried out using natural compounds extracted from plants against several microbial genera and/or species. Nanasombat and Lohasupthawee [17] studied the antimicrobial activity of extracts and essential oils from 14 spices tested against 20 serotypes of *Salmonella* and other members of the *Enterobacteriaceae* family, founding the following trend from greater to lesser degree of antimicrobial activity: Clove, cardamom, coriander, nutmeg, ginger, garlic, and basil among others. *E. coli* was the non-salmonellae strain more susceptible to most of the spice oils.

### 2.1. Onions and Garlic

The growth of many microorganisms is inhibited by onion and garlic. Several authors reported on the antimicrobial capacities of onions and garlic a long time ago [18,19,20,21,22,23,24]. Juices and vapours of these plants inhibit the growth of several microorganisms including bacteria (*Bacillus cereus*, *Clostridium botulinum*, *Escherichia coli*, *Lactobacillus*, *Salmonella*, *Staphylococcus aureus*, etc.) and fungi (*Aspergillus* spp., *Candida*, *Saccharomyces*, etc.) [23]. Conner et al. [18] reported that essential oils of onion (500 μg/mL) reduced the ethanol production by *Sacchraromyces cerevisiae*, suppressed the production of ethanol by *Hansenula anomala*, and delayed sporulation of *Lodderomyces elongisporus*. González-Fandos et al. [19] studied the inhibition of *S. aureus* growth and enterotoxin and thermonuclease production by garlic in brain heart infusion (BHI) broth. These authors found that *S. aureus* was inhibited at levels of 1.5% and over; enterotoxins A, B, and C1 were found with less than 1% of garlic, but at a 2% concentration the enterotoxin D was synthesized. Garlic inhibited thermonuclease production completely at levels greater or equal to 1.5%. Barone et al. [22] reported fungicidal activity of garlic extracts (68 µg/mL) against 39 of 41 clinical strains of *Candida albicans* in standing culture; the extract was fungistatic (50–300 µg/ mL) and fungicidal (>400 µg/mL) in shake culture. A very interesting fact found by Barone et al. [22] was a loss of antimicrobial activity against *C. albicans* when the garlic extract was heat treated at 37 °C, having food safety implications in culinary processes; moreover, the activity against the microorganism was stable under acidic conditions, but unstable under base conditions. Kim et al. [24] studied the activity of garlic and onion essential oils and their sulfides against several bacteria and yeasts: *S. aureus*, *E. coli*, *Enterobacter aerogenes*, *Leuconostoc mesenteroides*, *Pediococcus pentosaceus*, *Lactobacillus plantarum*, *Pichia membranefaciens*, *Saccharomyces cerevisiae*, *Candida utilis*, *Candida albicans*, *Zygosaccharomyces bisporus*, and *Zygosaccharomyces rouxii*. The minimum inhibitory concentrations (MIC) of garlic and onion oils, diallyl-trisulfide and -tetrasulfide, and dimethyl-trisulfide were 2–45 ppm for the yeasts studied; however, these compounds had weak activity against most of the bacteria (MIC > 300 ppm). The activity against the tested yeasts was not influenced by the storage or the pH.

### 2.2. Spices

As stated by Taylor and Davidson [23], spices are different parts (roots, seeds, leaves, fruits, etc.) of aromatic plants added as flavouring components to foods; among them, oregano, cinnamon, clove, and rosemary showed the greatest activity against microorganisms. Eugenol and cinnamic aldehyde are the major constituents of clove and cinnamon, respectively [23]. Cinnamon and cinnamic aldehyde have shown activity against bacteria (*Aeromonas hydrophila*, *Bacillus* spp., *Campylobacter jejuni*, verotoxin-producing *E. coli*, *Lactobacillus*, *Listeria monocytogenes*, *Salmonella*, *Shigella*, *S. aureus*, and *Streptococcus*) and fungi (*Aspergillus*, *Candida*, *Penicillium*, and *Saccharomyces*) [23,25,26,27,28,29,30,31]. Clove and eugenol are inhibitory to similar bacteria and fungi [28,29,30,31,32,33,34,35,36,37,38,39]. The antimicrobial effects of cinnamon alone or combined with potassium sorbate or sodium benzoate were tested against *Escherichia coli* O157:H7 at different temperatures in apple juice by Ceylan et al. [25]; the microorganism counts were reduced by approximately 2.0 log colony forming units (CFU)/mL at 8 or 25 °C by 0.3% cinnamon. Between cinnamon and the studied preservatives a synergistic activity was found: 0.3% of cinnamon combined with 0.1% of sodium benzoate or potassium sorbate killed 5 log CFU/mL in 11 or 14 d at 8 °C, respectively; the inhibitory effect was similar in 3 d by the same combinations at 25 °C. Thyme, oregano, dictamus, marjoram, lavender, rosemary, and sage were tested against *Penicillium digitatum* by Daferera et al. [26]; the growth and germination were inhibited by the essential oils of dictamus, marjoram, oregano, and thyme at 250–400 µg/mL, while lavender, rosemary, and sage were less effective. Friedman et al. [28] studied the activity of 119 essential oils against bacteria isolated from foods and clinical sources (*Campylobacter jejuni*, *E. coli* O157:H7, *L. monocytogenes*, and *Salmonella enterica*) founding that 39 oils were active against all four species of bacteria. Nielsen et al. [30] investigated the effect of spices and herbs oils and oleoresins against bread spoilage fungi (*Penicillium commune*, *P. roqueforti*, *Aspergillus flavus*, and *Endomyces fibuliger*) as an alternative to modified atmosphere packaging. Cinnamon, clove, garlic, and mustard had high activity, while oregano had weak activity against the growth of fungi; the more resistant microorganisms was *A. flavus*, and *P. roqueforti* the most sensitive.

The activities against the microorganisms of oregano and thyme have been assigned to carvacrol and thymol, respectively [23], showing activity against the bacteria *Aeromonas* spp., *B. cereus*, *Brochothrix thermosphacta*, *Campylobacter jejuni*, *Escherichia coli*, *Enterobacter faecalis*, *Lactobacillus plantarum*, *Listeria monocytogenes*, *Pediococcus cerevisiae*, *Pseudomonas*, *Proteus*, *Salmonella*, *Shigella*, *Staphylococcus aureus*, *Vibrio parahaemolyticus*, and *Yersinia enterocolitica* [7,34,40,41,42,43,44,45,46,47,48,49,50,51], and the moulds and yeasts *Aspergillus*, *Candida*, *Geotrichum*, *Penicillium*, *Pichia*, *Rhodotorula*, *Saccharomyces cerevisiae*, and *Schizosaccharomyces pombe* [52,53,54,55,56,57,58,59]. Burt and Reinders [7] quantified the antibacterial effect against *E. coli* O157:H7 of several essential oils with or without a stabilizer (such as agar) and an emulsifier (lecithin) at different temperatures. Oregano and thyme essential oils had the strongest properties; 0.05% of agar reinforced the activity of the essential oils at 10 °C, whereas the addition of 0.25% of lecithin reduced their activity. These authors reported that the combination of oregano or thyme with agar reduces the number of *E. coli* O157:H7 preventing its growth. Of 17 spices and herbs tested at 0.5–1%, only clove, basil, marjoram, oregano, rosemary, and thyme showed activity against *Shigella* spp. [34]. These authors combined temperature (12, 22, and 37 °C), pH (5.0, 5.5, and 6.0), NaCl (1–4%), and thyme or basil (0 or 1%), establishing that both can contribute as an inhibitory factor: *S. flexneri* did not grow for 7 d with basil and/or thyme, while growth was noted without them. The practical side of the study was the use of these spices in spaghetti sauce founding that, at 12 °C, the population of *S. sonnei* decreased after 16 d; the population did not reduce its counts at 4 °C. Seaberg et al. [41] addressed the fact that different batches of the same plant species have a genetic heterogeneity that represents a problem for their use against microbial growth and for achieving the “clean label” for the food industry. To overcome the situation, a clonal line of oregano was isolated, and its ethanol extracts together with its main constituents—thymol and carvacrol—were used in both broth and meat systems to study its activity against *Listeria monocytogenes*; all thymol and carvacrol (150–200 ppm) and the clonal line (1200 ppm) inhibited the *L. monocytogenes* growth in both systems. Singh et al. [42] also evaluated the activity of essential oils from different plants against *Listeria monocytogenes* in peptone water and hotdogs, finding that thyme and clove (1 mL/L) were highly effective inhibiting the population of *L. monocytogenes* below detection limits. Carvacrol was also investigated by Ultee et al. [43] for its effect on *Bacillus cereus* and the production of diarrheal toxin; its counts were reduced with concentrations of about 0–0.06 mg/mL in BHI broth—an 80% decline in toxin production was detected with 0.06 mg/mL. Carvacrol, thymol, cymene, and terpinene were studied against *E. coli* O157:H7 by Burt et al. [45] and Kiskó and Roller [47]; carvacrol and thymol were additive in combination showing bacteriostatic and bactericidal activities (1.2 mmol/L), and cymene and terpinene did not show antibacterial activity up to 50 mmol/L. The inhibitory activity of several natural compounds (thymol, carvacrol, eugenol, cinnamic acid, and diacetyl) alone or in combination with nisin against *E. coli* and *Salmonella enterica* serovar Typhimurium [48], or *Bacillus subtilis* and *Listeria innocua* [49] was studied. Nisin alone showed no antibacterial activity. Thymol was the most effective with concentrations of 1.0–1.2 mmol against *S. enterica* and *E. coli*; the combination of nisin showed no improvement of the antimicrobial activity. All the organic compounds exhibited activity against the Gram-positive microorganisms with concentrations between 0.8 and 15.0 mM; the interaction between the organic compounds and nisin showed different patterns, varying from synergistic (carvacrol, eugenol, or thymol; nisin plus cinnamic acid only against *L. innocua*) to antagonistic (nisin plus diacetyl). The anticandidal activity of the major phenolic compounds of oregano (carvacrol at 0.1%) and clove (eugenol at 0.2%) essential oils was studied by Chami et al. [53]. Both compounds were fungicidal in exponentially growing *Candida albicans*. Also using *Candida albicans*, the activity of origanum, carvacrol, nystatin, and amphotericin B were tested by Manohar et al. [57]. *C. albicans* growth was completely inhibited by origanum oil at 0.25 mg/mL; origanum oil and carvacrol inhibited both germination and mycelial growth in a dose-dependent manner.

Sage (containing thujone) and rosemary (with borneol, pinene, camphene, and camphor) also have antimicrobial activity [23,44]. Oregano, thyme and savoury [7], and sage and rosemary [60,61] essential oils showed pronounced bactericidal properties against *E. coli* O157:H7 and other foodborne pathogens. Pirbalouti et al. [62] reported antibacterial activity against *L. monocytogenes* by several plant extracts including essential oils from *Thymus* spp. In contrast, other authors [42,54,59,63] have found that essential oil of spices had little antimicrobial activity against bacteria and yeasts may be due to the assays utilized [23]. 

Several other spice essential oils have shown potential for antibacterial and antifungal activity. Sweet basil demonstrated activity against fungi such as *Mucor* and *Penicillium* although little activity against bacteria [23,64]; the main agents are linalool and methyl chavicol [23]. Essential oils from different varieties of sweet basil were tested for their activity against Gram-positive and Gram-negative foodborne bacteria, yeasts, and moulds by Lachowicz et al. [64]; all basil’s essential oils showed activity against the microorganisms tested with the exception of *Flavimonas oryzihabitans* and *Pseudomonas* spp. Vanilla beans have vanillin as their major constituent, being most active against moulds and Gram-positive bacteria [23,65]. Delaquis et al. [65] studied the activity of vanillin and vanillic acid against *Listeria monocytogenes*, *L. innocua*, *L. grayi*, and *L. seeligeri*. All strains were inhibited by concentrations of about 23–33 mM; concentrations of about 100 mM vanillic acid at pH > 6.0 was not effective against the microorganisms, but with 10 mM at pH 5.0 the inhibition was complete. A declining pH increased the lethal activity of vanillic acid, and vanillin plus vanillic acid gave additive inhibitory effects.

Other essential oils from spices have potential antimicrobial activity as well as antifungal, such as cilantro-also known as coriander, fingerroot, lemongrass, savory, and tea tree oil [23,28,44,58,61,66,67,68,69,70].

### 2.3. Cruciferae 

Cabbage, cauliflower, broccoli, Brussels sprouts, horseradish, kale, kohlrabi, mustard, turnips, and rutabaga are members of this family. Isothiocyanates are reported as antimicrobial agents [23] against bacteria (*E. coli* O157:H7, *L. monocytogenes*, *Salmonella*, *S. aureus*, *Serratia*, *Lactobacillus sake*, *Pseudomonas*, and *Enterobacteriaceae*) [71,72,73] and fungi and yeast (*Penicillium expansum*, *Aspergillus flavus*, and *Botrytis cinereal*) [74].

Delaquis et al. [71] and Ward et al. [72] tested the behaviour of bacteria in pre-cooked roast beef with vaporized horseradish essential oil at 4 °C for 28 d. *Pseudomonas* spp. and some members of the family *Enterobacteriaceae* were inhibited; lactic acid bacteria were more resistant. Interestingly, the colour of the cooked meat was preserved in samples stored with horseradish essential oil. The growth of *S. aureus*, *E. coli* O157:H7, *S. typhimurium*, *L. monocytogenes*, and *Serratia grimesii* was inhibited at 12 °C for 7 d of storage under aerobic conditions.

### 2.4. Phenolic Compounds

Monophenols, diphenols, and triphenols are simple phenolic compounds. The use of wood smoke for food preservation implies the use of simple phenols (cresol, hydroquinone, gallic acid); additionally, their use gives a desirable flavour [23]. Liquid smoke is a method widely used in cheese surface inhibiting the growth of fungi such as *Aspergillus oryzae*, *Penicillium camemberti*, and *Penicillium roqueforti* [75]; these authors found that only isoeugenol inhibited all these molds. Cresol (forms m- and p-) slightly inhibited the growth of *P. camemberti*, and guaiacol, 4-methylguaiacol, and m- and p-cresol inhibited the growth of *A. oryzae*.

The phenolic acids are present in plants and can inhibit bacteria such as *Aeromonas hydrophila*, *E. coli*, *E. faecalis*, *Salmonella* serovar Enteritidis, *L. monocytogenes*, and *S. aureus*, [23,76]. The most effective compound was the phenolic antioxidant tertiary butylhydroquinone with MIC of 64 μg/mL [76].

Hydroxycinnamic acids (such as caffeic, coumaric, ferulic, and sinapic acids) have different inhibition effects against *B. cereus* and *S. aureus*; *P. fluorescens* and *E. coli* are more resistant to them [23,77]. It has been reported the antifungal properties of hydroxycinnamic acids, i.e., inhibiting the production of aflatoxins from *A. flavus* and *A. parasiticus* [23,78]. Herald and Davidson [77] reported the antibacterial activity of caffeic, ferulic, and p-coumaric acids against *Escherichia coli, Staphylococcus aureus,* and *Bacillus cereus*; p-coumaric acid was the most effective against *E. coli* with concentrations of about 1000 μg/mL at pH 5.0 for 48 h, and *S. aureus* and *B. cereus* with concentrations of about 500 μg/mL at pH 5.0 for 48 h or at pH 7.0 for 9 h, respectively. Inhibition increased as pH decreased with *E. coli* and *S. aureus* but not *B. cereus*. Chipley and Uraih [78] studied the antimicrobial activity of o-nitrobenzoate, p-aminobenzoate, ethyl aminobenzoate, ethyl- and methyl-benzoate, salicylic acid, trans-cinnamic acid, trans-cinnamaldehyde, ferulic acid, o-acetoxy benzoic acid, and anthranilic acid on *Aspergillus flavus* and *A. parasiticus* growth and aflatoxin production at 27 °C. Both methyl- and ethyl-benzoate were the most effective at concentrations of about 2.5–5.0 mg/25 mL of medium reducing the mycelial growth and the aflatoxin production.

Furocoumarins are present in carrots, celery, citrus fruits, parsley, and parsnips. Several authors reported their antimicrobial activity against *E. coli* O157:H7, *Erwinia carotovora*, *L. monocytogenes*, and *Micrococcus luteus* [23,79]. The antimicrobial activity of furanocoumarins against *L. monocytogenes*, *E. coli* O157:H7, and *Micrococcus luteus* was investigated in a model food system (25% commercial vegetable baby food in peptone water) by Ulate-Rodríguez et al. [79]. The growth of *L. monocytogenes* was inhibited with lime peel extract and cold-pressed lime oil, but not the growth of *E. coli* O157:H7; *M. luteus* counts were inhibited only by the cold-pressed lime oil. The minimum inhibitory and the minimum bactericidal concentrations of *L. monocytogenes* were 32 or 43 μg/g, respectively.

Flavonoids, such as catechins, flavons, flavonols, and their glycosides, are present in apples, barley, grapes, plums, sorghum, and strawberries [23,80]. Cushnie and Lamb [80] reported antifungal, antiviral, and antibacterial activity. Quercetin activity was attributed to the inhibition of the enzyme DNA gyrase; the inhibition of the cell membrane functions by the activity of sophoraflavone G and (-)-epigallocatechin gallate was reported and, moreover, the inhibition of the energy metabolism by the licochalcones A and C [80]. Other studied flavonoids are 2,4,2′-trihydroxy-5′-methyl chalcone, apigenin, galangin, lonchocarpol A, myricetin, robinetin, and rutin.

### 2.5. Hops

The hop (*Humulus lupulus* L.) flower’s resin is used in the brewing industry for the bitter flavour it gives to beer [23]. Hop contains compounds (prenylated acylphloroglucinols and xanthohumol) that have inhibited bacteria growth, mostly Gram-positive [81,82,83,84,85,86,87]. The use of bitter acids as antimicrobials was approved by the Food Safety and Inspection Service (FSIS), USA [88]. Kramer et al. [81] studied the effect of hop extracts against some pathogens related with food using in vitro and meat model applications at 2 and 8 °C. The MIC of hop extracts with bitter acids (α- and β-acids) or xanthohumol were tested against *E. coli*, *S. aureus*, *S. enterica*, and *L. monocytogenes*. The xanthohumol and the β-acid inhibited the growth of the Gram-positive bacteria (MICs of 12.5 and 6.3 ppm, respectively), and the α-acid was less active (MIC of 200 ppm); in contrast, the Gram-negative bacteria were highly resistant. These authors concluded that “hop extracts could be used as natural preservatives in food applications to extend the shelf life and to increase the safety of fresh products.” Bogdanova et al. [82] investigated the antibiofilm properties of hop compounds (humulone, lupulone, and xanthohumol) against *Staphylococcus* spp., including strains that were methicillin-susceptible and resistant. All compounds showed antimicrobial activity against all strains; lupulone, followed by xanthohumol had the strongest effect. Lupulone and xanthohumol penetrated the biofilm reducing the number of cells or reducing completely their number at the higher concentrations (lupulone: 125 μg/mL; xanthohumol: 60 μg/mL). Hop extracts showed different grades of inhibition against *L. monocytogenes* in food [86]: In coleslaw, 1 mg/g of hop extract increased the inactivation; in milk, 0.1–1 mg/mL was inhibitory; and in cottage cheese, hop extract was bactericidal at 0.1–3 g/kg. These authors concluded that, overall, the activity against *L. monocytogenes* in food was enhanced with acidity and lower fat content.

Some fungi are inhibited by hop acids [89,90] as well as protozoa [91]. Mizobuchi et al. [89] isolated a new flavonone (6-isopentenylnaringenin) from hard resins of hops; it was tested together with xanthohumol and isoxanthohumol showing antifungal activities against *Candida albicans*, *Fusarium oxysporum*, *Trichophyton mentagrophytes* and *T. rubrum*, and *Mucor rouxianus*. Srinivasan et al. [91] studied the antimicrobial spectrum of hop acid components for antiprotozoal activity, founding that ciliated protozoa were more sensitive than amoebae; plasmodia were sensitive but at a lower level than to the anti-malarial drugs. Xanthohumol was particularly potent, and the effect was enhanced by carbon dioxide.

### 2.6. Other Plants

Ahn et al. [92] focused their studies on the extracts from grape seed and pine bark; these authors found that their extracts can be used against *E. coli* O157:H7, *S. enterica* serovar Typhimurium, and *L. monocytogenes* in vitro and ground beef. The populations of *E. coli* O157:H7, *S. enterica* serovar Typhimurium, and *L. monocytogenes* decreased below 10 CFU/mL after 16 h. Markin et al. [93] studied olive leaves extracts founding deadly effects on bacteria, dermatophytes, and yeast. Olive leaf 0.6% extract killed within 3 h almost all cells from cultures of *E. coli*, *B. subtilis*, *Klebsiella pneumoniae*, *Pseudomonas aeruginosa*, and *S. aureus*; 1.25% after 3 d inhibited the growth of dermatophytes such as *Microsporum canis*, *Trichophyton mentagrophytes* and *T. rubrum*, whereas 15% of plant extract killed after 24 h of incubation all the cells from the yeast *Candida albicans*. Dogasaki et al. [94] and Ibrahim et al. [95] mentioned the antibacterial properties of coffee and its compounds such as caffeic acid, chlorogenic acid, and protocatechuic acid; these compounds inhibited the growth of *Legionella pneumophila* and *E. coli* O157:H7, respectively. Furthermore, tea (*Camellia sinensis*) was also demonstrated to feature antimicrobial properties [96,97,98] through its predominant catechin, epigallocatechin gallate, against methicillin-resistant *S. aureus*. Shan et al. [99] reported the activity of cinnamon stick extracts (*Cinnamomum burmanii* Blume) against *B. cereus*, *E. coli*, *L. monocytogenes*, *S. aureus*, and *Salmonella anatum*. Major compounds in the cinnamon stick were identified: E-cinnamaldehyde and polyphenols; both components significantly contributed to the antimicrobial properties.

### 2.7. Plant By-Products

Large amounts of by-products are generated during the food processing of plants, such as fruit pomace, husks, kernels, peels, pulps, seeds, and unused flesh [2]. Usually considered as a waste, these by-products possess bioactive compounds with antimicrobial activity being promising sources for their commercial exploitation; Gyawali and Ibrahim [2] list some plant by-products as antimicrobials.

Extracts of grape pomane [100] and olive pomace [101,102] have shown to be able to inhibit the growth of *E. coli*, *Enterobacter* spp., *S. aureus*, *Salmonella* spp., and *L. monocytogenes*, and other spoilage and pathogenic bacteria. Sagdic et al. [100] incorporated grape pomace extracts into beef patties at different concentrations: 1–10% for 12, 24 and 48 h. All the microorganisms tested (*Enterobacteriaceae* and spoilage microorganisms) were inhibited at a concentration of 10% in all the storage periods. Friedman et al. [101] evaluated the bactericidal activity of 10 food-based powders against *E. coli* O157:H7, *S. enterica*, *S. aureus*, and *L. monocytogenes*. Olive pomace, juice powder, and leaves were active against all bacteria. All powders had strong activity against *S. aureus*.

Fruit peels are also important. Pomegranate fruit peels extracts showed their antimicrobial activity enhancing the shelf-life of chicken products [103], and their ability to inhibit the growth of *E. coli, B. cereus, L. monocytogenes, S. aureus,* and *Y. enterocolitica* [104,105,106]. Pomegranate peel showed good activity against *S. aureus* and *B. cereus* (MIC of 0.01%); concentrations of 0.1% inhibited *Pseudomonas* but *E. coli* and *S. typhimurium*. The shelf life of chicken products was enhanced by 2–3 weeks with the addition of pomegranate peel during chilled storage [103]. Li et al. [105] investigated the activity of the tannin-rich fraction from pomegranate rind against *L. monocytogenes*; punicalagin and ellagic acid were detected, and the MICs against *L. monocytogenes* strains were 1.25–5.0 mg/mL. The same research group [106] evaluated the effects of the same fraction on both the virulence gene expression and the *L. monocytogenes* interaction with the epithelial cells. The adhesion to and the invasion of Caco-2 cells were reduced at 2.5 mg/mL. Guava, jackfruit, mango, papaya, plum, tamarind, and their seeds were effective against *B. subtilis*, *E. coli*, *S. aureus*, and *P. aeruginosa* [107]. A major fruit by-product is tomato seeds from the tomato processing industry; tomato seeds extracts have shown inhibition of Gram-positive bacteria and fungi [108]. These authors studied the antimicrobial potential of tomato seed extracts against Gram-positive (*Bacillus cereus*, *Enterococcus faecalis*, *Micrococcus luteus*, *Staphylococcus aureus* and *S. epidermidis*) and Gram-negative (*E. coli*, *Proteus mirabilis*, *Pseudomonas aeruginosa*, and *S. typhimurium*) bacteria and fungi (*Aspergillus fumigatus*, *Candida albicans*, and *Trichophyton rubrum*). *E. faecalis* was the most susceptible Gram-positive bacteria (MIC of 2.5–10 mg/mL). *C. albicans* was the most susceptible fungal species (MIC of 5–10 mg/mL).

Coffee husks, peel, and pulp are some of the main by-products obtained from coffee processing industry [2,109,110]; extracts from these by-products contain large amounts of phenolic compounds (tannins, flavonols, flavandiols, flavonoids, and phenol acids) and are potential natural preservatives for food [2]. Quoting Taveira et al. [108] and Gyawali and Ibrahim [2], “the waste produced by the food-processing industry could be incorporated into antimicrobial packaging or utilized as edible antimicrobial films”.

## 3. Natural Antimicrobials from Animals

Some of the animal defence mechanisms have antimicrobial properties [1,23] destroying the cell membranes [1,60] and killing both Gram-negative and -positive bacteria [1].

### 3.1. Peptides

Antimicrobial peptides from animal origin have a broad range of antibacterial activities as well as antiviral [111].

Pleurocidin is a peptide with antimicrobial activity found in the skin secretions of the winter flounder (*Pleuronectes americanus*) [112], and it is active against Gram-positive and -negative bacteria such as *E. coli* O157:H7, *L. monocytogenes*, *Saccharomyces cerevisiae*, *Penicillium expansum*, and *Vibrio parahemolyticus* [113,114]. Burrowes et al. [113] evaluated pleurocidin in food applications using 18 microbial species. Pleurocidin was effective against *E. coli* O157:H7, *L. monocytogenes*, *P. expansum*, *S. cerevisiae*, and *V. parahemolyticus* with MIC of 5.3, 23.0, 20.6, 5.5, and 69 µM, respectively; no haemolytic or cytotoxic effect on intestinal cells were found. Patrzykat et al. [114] identified peptide effects studying a flounder pleurocidin and frog dermaseptin hybrid. At 2 µg/mL, dermaseptin inhibited the growth of *E. coli* but did not kill the cells within 30 min; concentrations equal to or higher than 20 µg/mL reduced the viable counts by 2 log within 5 min. Pleurocidin showed variations of this antimicrobial pattern.

Other antimicrobial peptides are defensins, protamine, magainin, and casocidin [1]. Defensins are produced by vertebrates-phagocytes of mammals and epithelial cells, with antimicrobial properties against bacteria and fungi, as well as viruses [60]. Protamine and magainin are active against bacteria and fungi [60,115]: protamine is a protein obtained from sperm cells of vertebrates [116], and magainin from the skin of the frog *Xenopus laevis* [117,118]. Protamine is a cationic peptide; its activity against microorganisms is probably due to its electrostatic affinity to negatively charged bacteria. Potter et al. [115] tested this hypothesis in model broth (tryptic soy broth) and food systems (milk and ground beef). The analysis of 21 bacteria revealed that the most negatively charged were also the most susceptible. Kim et al. [116] investigated the suppressive effects of protamine on the growth of oral pathogens; 12 strains of streptococci, *Actinomyces naeslundii* and *A. odontolyticus*, *Aggregatibacter actinomycetemcomitans*, *Candida albicans*, *Enterococcus faecalis*, *Fusobacterium nucleatum*, *Lactobacillus acidophilus*, and *Porphyromonas gingivalis* were inhibited (MIC of 0.009–20 mg/mL). Zasloff [117] tested magainin for its antibacterial activity founding that, at low concentrations, inhibited the growth of numerous species of bacteria (*E. coli*, *Enterobacter cloacae*, *Klebsiella pneumoniae*, *Staphylococcus epidermidis*, *Staphylococcus aureus*, *Citrobacter freundii*, *Pseudomonas aeruginosa*, *Pseudomonas putida*, *Serratia marcescens*, *Proteus mirabilis*, and *Streptococcus fecalis*) and fungi (*Saccharomyces cerevisiae*, *Cryptococcus neoformans*, and *Candida albicans*) and induce osmotic lysis of protozoa (*Amoeba proteus*, *Euglena gracilis*, and *Paramecium caudatum*). Casocidin is another peptide obtained from bovine milk with antibacterial activity against *E. coli* and *Staphylococcus carnosus* [119]. The primary structure of casocidin is a fragment of 39 amino acids of bovine αs2-casein. The casein-αs2 is not present in human milk, so Zucht et al. [119] hypothesized that “these findings could explain the different influence of human and bovine milk on the gastrointestinal flora of the suckling.”

Lactoferrin is a peptide with capacity against Gram-positive and -negative bacteria, fungi, and parasites [2,120,121]. Murdock et al. [120] determined whether nisin and lactoferrin would act synergistically against *L. monocytogenes* and *E. coli* O157:H7. *L. monocytogenes* was inhibited with 1000 μg/mL of lactoferrin, although *E. coli* O157:H7 counts initially decreased and then recovered to cell counts similar to the control. Lactoferrin (500 μg/mL) plus nisin (250 IU/mL) effectively inhibited the *E. coli* O157:H7 growth, whereas 250 μg/mL plus 10 IU/mL, respectively, had an inhibitory effect suggesting that lactoferrin and nisin act synergistically to inhibit both microorganisms. López-Expósito et al. [121] studied whether the antimicrobial activity of nisin could be enhanced by lactoferrin f and αs2-casein f against *Escherichia coli*, *Listeria monocytogenes*, *Salmonella choleraesuis*, and *Staphylococcus epidermidis*. Results showed a synergistic effect against *E. coli* and *S. epidermidis*; moreover, another synergistic effect was found between αs2-casein f and nisin against *L. monocytogenes* because of its capacity to develop resistance to nisin. Lactoferrin binds iron [1,2] and is used as antimicrobial in meat products [122]. Murdock et al. [120] and Al-Nabulsi and Holley [123] reported the antimicrobial activity of lactoferrin against foodborne bacteria such as *E. coli*, *Carnobacterium* spp., *Klebsiella*, and *L. monocytogenes*. Lactoferrin in a concentration of about 8 mg/mL killed 4 log CFU/mL of *Carnobacterium viridans* at 4, 10 and 30 °C and neutral pH in a broth system [123].

Lactoperoxidase is a protein (glycoprotein enzyme) present in raw milk, colostrum, saliva, and other secretions [23,124]. Lactoperoxidase reacts with thiocyanate and hydrogen peroxide forming the termed lactoperoxidase system (LPS) with antimicrobial capacities. Thiocyanate is found in milk and other animal secretions by the metabolism of amino acids and glucosides from the diet or by detoxification of thiosulfates. Hydrogen peroxide is not present in milk, so it needs to be added, or obtained from lactic acid bacteria activity or enzymatic action [23,124,125]. Potential mechanisms used by the LPS were reviewed by Bafort et al. [125]. The LPS inhibits both Gram-positive and -negative pathogens including *E. coli* O157:H7, *Y. enterocolitica*, *Salmonella*, *S. aureus*, *L. monocytogenes*, *C. jejuni* and *P. aeruginosa* in different foods [23,124,126,127,128,129,130]. Elliot et al. [127] assessed the growth of *E. coli* O157:H7, *L. monocytogenes*, *S. aureus*, *S. enterica* subsp. enterica serovar Typhimurium, *Pseudomonas aeruginosa*, *Yersinia enterocolitica*, and beef microbiota on meat surfaces treated with the LPS at 37 °C for 24 h, 12 °C for 7 d, 12 to −1 °C for 1 week, and −1 °C for 4 weeks. LPS was more effective at refrigeration temperatures, inhibiting the growth of *Pseudomonas* from beef microbiota but of lactic acid bacteria. McLay et al. [128] evaluated an LPS-monolaurin system (5–200 mg/kg of lactoperoxidase and 50–1000 ppm of monolaurin) for the inhibition of *E. coli* O157:H7 and *Staphylococcus aureus* in growth, milk and ground beef. In broth, the growth of both microorganisms was inhibited more strongly than in milk, and in milk more than in ground beef. The potential use of the LPS in broth at 37 °C and ground meat preparations at 0, 6, and 12 °C was examined by Kennedy et al. [129]. *L. monocytogenes* was the most sensitive, followed by *Staphylococcus aureus* and *E. coli* O157:H7. The inhibition was highly dependent of the temperature: it was maximal at a temperature adequate but not optimal for the bacterial growth. The LPS was tested against *Salmonella enteritidis* in tomato and carrot juices, milk, liquid whole egg, and chicken skin extract under different conditions [130]; at low pH and 30 °C, LPS was more effective in vegetables than in animal products.

Avidin is present in egg albumen and stable to heat and pH [23]. Avidin is a glycoprotein that binds biotin, creating one of the strongest non-covalent interactions in nature [131]. Biotin probably plays a role in the immune system because it is an enzyme cofactor in the tricarboxylic acid cycle and in the biosynthesis of fatty acids [132,133,134]. Avidin has been extensively used for biochemical assays, diagnosis, and drug delivery [135]. Recently, there has been growing interest in studying this avidin-biotin interaction in nanoscale drug delivery systems [131,136]. Korpela et al. [137] studied the binding of avidin to Gram-negative and -positive bacteria (*Enterobacter cloacae*, *E. coli*, *B. cereus*, *P. aeruginosa*, *Serratia marcescens*, *Klebsiella pneumoniae*, *Streptococcus pyogenes*, and *Staphylococcus aureus* and *S. epidermidis*). Avidin showed binding capacity to all the tested Gram-negative bacteria and to some Gram-positive. The *E. coli* K-12 avidin receptor was the outer membrane’s porin protein (OmpFOmpC); these authors hypothesised that avidin traps biotin preventing its entry into the cell. Moreover, the avidin binding to the cell membrane may also imply a role in the mechanism of infection.

Ovotransferrin or conalbumin is another glycoprotein that also occurs in egg albumen and with inhibitory activity against both Gram-positive and -negative bacteria, and some yeasts [23,138,139,140]. Bacterial sensitivity to ovotransferrin varied among species, being *E. coli*, *Pseudomonas*, and *Streptococcus mutans* the most sensitive, and *Staphylococcus aureus, Proteus*, and *Klebsiella* the more resistant [138]. Although ovotransferrin’s antimicrobial property is thought to be the result of its capacity to bind the iron used by the microorganisms for growth, recent studies suggest that its role is also iron independent [138,139]. Ovotransferrin properties imply applications such as ingredients for infant formula, food additive, or antimicrobial agent for animal health [139,141]. Furthermore, ovotransferrin has similarities to the homologous mammalian lactoferrin in terms of its protective roles, suggesting a “direct relationship between egg consumption and human health” [141].

Lysozyme is an enzyme found in egg white and milk, and even in blood [1,142,143,144,145]. Masschalck and Michiels [146] reviewed the properties against the microorganisms as well as the mode of action of lysozyme against Gram-positive and -negative bacteria, also providing insight into the causes of bacterial resistance. The antimicrobial capacities of lysozyme against bacteria are mainly due to its enzymic activity; lysozyme acts through peptidoglycan hydrolysis and cell lysis [1,143]. However, a new nonlytic activity has been shown by new findings [146,147]. As Gram-negative bacteria are resistant to lysozyme because of their outer membrane, different strategies were developed to extend its spectrum, including denaturation, modification (by attachment of polysaccharides, fatty acids, etc.), genetic modification, membrane permeabilizing agents (ethylenediaminetetraacetic acid, EDTA), and/or permeabilizing treatments such as high hydrostatic pressure treatments [146,148,149,150,151,152,153,154,155]. In order to study the role of the lysozyme enzymatic activity on its capacity against Gram-positive bacteria (*S. aureus* and *Bacillus subtilis*), Ibrahim et al. [148] constructed an inactive mutant of lysozyme. These authors revealed that the lysozyme activity against Gram-positive bacteria is independent of its muramidase activity; thus, the antibacterial action is due to structural factors.

### 3.2. Polysaccharides

Chitosan is produced commercially from chitin, a by-product obtained from exoskeletons of crustaceans and arthropods [1,23], with capacity to inhibit the growth of moulds and yeasts (*Aspergillus flavus*, *Botrytis cinerea*, *Byssochlamys* spp., *Mucor racemosus*, *Rhizopus stolonifer*, *Saccharomyces cerevisiae*, and *Zygosaccharomyces bailii*) and bacteria (*E. coli*, *Lactobacillus fructivorans*, *L. monocytogenes*, *Salmonella*, *S. aureus*, and *Y. enterocolitica*) from food [150,156,157,158,159,160,161,162,163,164]. Oh et al. [156] tested the antimicrobial activities of chitosan against food spoilage microorganisms in mayonnaise (*Lactobacillus plantarum* and *L. fructivorans*, *Serratia liquefaciens*, and *Zygosaccharomyces bailii*) founding an important decrease of the counts of *L. fructivorans* and *Z. bailii* at 25 °C. Roller and Covill [158] studied the use of chitosan (3 g/L) in mayonnaise with 0.16% of acetic acid or 1.2–2.6% of lemon juice and a population of ca. 5–6 log CFU/g of *S. enteritidis*, *Z. bailii*, or *L. fructivorans* at 5 or 25 °C for 8 d; the results of these authors showed that chitosan with acetic acid could be used as a natural preservative under such conditions. Chitosan at a concentration of 0.005% combined with sodium benzoate (0.025%) were synergic against yeasts (*Saccharomyces exiguus*, *Saccharomycodes ludwigii* and *Torulaspora delbrueckii*) in saline solutions [159]. Recently, chitosan’s films with antimicrobials attached (garlic oil, sorbic acid, and nisin) to the polymer were used for packaging applications or in combination with ethanolic extract and polypropylene in order to contact the surface of the food; these films inhibited the growth of *E. coli*, *Cronobacter sakazakii*, *Salmonella*, *Staphylococcus*, *L. monocytogenes*, and *B. cereus* [150,158,165,166,167,168,169,170,171]. Sagoo et al. [159] and Devlieghere et al. [167] theorized that chitosan may interact with the cytoplasmic membrane’s anionic polysaccharides and/or interfere the cell protein synthesis, both resulting in cell inhibition by altered permeability and/or compromised protein transport. The molecular weight of chitosan, its role as water binding agent and enzyme inhibitor, the improvement of membrane permeability, its activity as a bio-absorbent competing against bacteria nutrients, and its capacity to bind to DNA and inhibit the synthesis of mRNA and proteins have been reported [163,172,173,174,175,176,177,178,179].

### 3.3. Lipids

Food lipids may inhibit the proliferation of microorganisms [1], e.g., those present in milk can inhibit Gram-positive and -negative bacteria, and fungi [180,181,182,183,184,185]. The activity of triglycerides and lipids from bovine milk was investigated against *E. coli* O157:H7, *S. enteritidis*, *Campylobacter jejuni*, *L. monocytogenes*, and *Clostridium perfringens* by Sprong et al. [180]. C10:0 and C12:0 fatty acids and sphingolipids showed bactericidal activity, whereas phosphoglycerides were moderately bactericidal. These authors [181] also studied the activity of sphingolipids combined with C10:0 and C12:0 fatty acids, and unsaturated C18 fatty acids; *L. monocytogenes* and *Campylobacter jejuni* were very sensitive, whereas *E. coli* O157:H7 and *S. enteritidis* were less vulnerable. Eicosapentaenoic acid and docosahexaenoic acid, two fatty acids from animal origin, can inhibit the growth of bacteria [186,187,188]. The knowledge about their mode of action is limited; their first action is the electron transport chain’s disruption and the cell membrane’s oxidative phosphorylation [188]. Furthermore, the mechanism of action also implies the inhibition of the enzymatic activity by the cell, the damage of the nutrient uptake, or peroxidation. The combination of fatty acids and monoglycerides was studied showing an increase in the antimicrobial activity [1]. Their broad spectrum of activity makes them very useful as agents against microorganisms for various applications in food safety increasing food quality through its preservation [188].

## 4. Natural Antimicrobials from Bacteria and Viruses: Biopreservation

Biopreservation is defined as “the use of microorganisms (including bacteriophages), their metabolic products, or both to preserve foods that are not generally considered fermented” [189].

### 4.1. Controlled Acidification

The production of acid by lactic acid bacteria (LAB) under controlled acidification conditions is a preservation form very important in food production; the pH of the food, the characteristics of the targeted microorganism, the fermented carbohydrate that is going to be used by the biopreservatives, the LAB growth kinetics, the temperature (refrigeration or abuse), and the targeted pathogens are factors to be taken into account [189]. The use of LAB for biopreservation comes back to the 1950s for preventing the production of botulinum toxin [190,191,192]; *Clostridium botulinum* is unable to grow at pH < 4.6, so LAB is added to the food for acid production after the use of a fermentable carbohydrate also added to it and, consequently, the pH decreases [190,193]. Hutton and Chehak [193] described the “Wisconsin process” (lactic acid starter culture combined with sucrose) as a similar method to be applied in bacon with a reduced content of nitrite. These authors also found a similar effect with the combination of *Pediococcus acidilactici* plus dextrose in chicken salad; it was interesting to report that conditions of abuse temperature were the most effective for the production of acid by *P. acidilactici* after the use of dextrose.

### 4.2. Bacteriocins

Some lactic acid bacteria produce ribosomally synthesized peptides, called bacteriocins, with antimicrobial capacity that are not lethal to the host; these proteins inhibit both pathogenic and spoilage bacteria without changing the physicochemical characteristics of the food, that is, the inhibition is not exerted by acidification, protein denaturation, or other processes [189,194,195,196,197,198,199,200,201,202,203,204]. The interest in LAB-produced bacteriocins has grown dramatically because of their antimicrobial capacities and the use of LAB as starter cultures [198]. The use of bacteriocins or bacteriocinogenic LAB or both is important to the food industry because of the demand for natural products by consumers that are also increasing concern about foodborne pathogens [189].

Gram-positive bacteria are inhibited by most bacteriocins. Gram-negative bacteria can be increasingly sensitive to bacteriocins after the use of chelating agents or hydrostatic pressure [205,206]; these techniques can also be synergic enhancing the action of bacteriocins against Gram-positive bacteria [189,207], or even with the use of nanoparticles [208,209] or nanovesicles [210].

Bacteriocins can be used in different ways in foods [189]: (i) They can be directly added to foods inhibiting the growth of both pathogenic and spoilage bacteria; nisin is the only bacteriocin commercially available. Nisin is added to milk, cheese, canned foods, mayonnaise, and other foods. It is considered as a Generally Recognized as Safe (GRAS) food preservative, and once it is absorbed onto surfaces it inhibits the growth of *Listeria* spp. and prevents the formation of biofilms [211,212,213,214,215,216]. Pediocins also inhibit the growth of *L. monocytogenes* and they are used in salads and salad dressings, cream, cheese and meats [216,217,218,219,220,221,222,223,224,225]. Reuterin is secreted by *Lactobacillus reuteri* and possess activity against Gram-positive and -negative pathogenic bacteria [226,227]. (ii) To add bacteriocin-producing bacteria to non-fermented foods or use them as starter cultures for the improvement of food safety. For a fine description see Montville and Chikindas [189]. Natamycin is produced by fermentation using *Streptomyces* spp. acting against foodborne moulds and yeasts; however, it is inactive against bacteria and viruses [1,228]. (iii) A third way is to use bacteriocin-producing bacteria as starter cultures to direct the fermentation. The benefits of defined starter cultures depend on their capacity to predominate over the indigenous microbiota [229,230,231,232,233].

An increasing problem is resistance to bacteriocins. The emergence of pathogens resistant to bacteriocins can undermine their use as antimicrobial agents. For example, nisin-resistant isolates have been generated from *C. botulinum*, *L. monocytogenes*, *S. aureus*, and *Bacillus licheniformis*, *B. subtilis*, and *B. cereus* [234,235,236,237,238]. Ming and Daeschel [234] evaluated Gram-positive pathogenic and food spoilage bacteria for their resistance to nisin obtaining a *L. monocytogenes* mutant (resistant to nisin at 2000 U/mL). The resistant mutant had straight-chain fatty acids at a higher percentage, and less percentage of branched-chain fatty acids; thus, both the cell membrane structure and function suffered changes as a resistance response to nisin. Turovskiy et al. [235] investigated the quorum sensing mediated by the autoinducer AI-2 as a mechanism for triggering the stress response in *L. monocytogenes*; thus, they examined the acquisition of resistance to nisin and lactic acid by the microorganism. After pre-exposing the cells to the autoinducer and being challenged with specific stresses, the resistance to nisin and lactic acid was not mediated through quorum sensing. The frequencies of colony formation on agar media with different concentrations of nisin by different strains of *Clostridium botulinum* were determined by Mazzotta et al. [236]. Increasing concentrations of nisin generated resistant isolates, and the cells’ nisin resistance was maintained by their spores. Naghmouchi et al. [237] developed variants of *Listeria monocytogenes* resistant to pediocin PA-1, divergicin M35, and nisins A and Z. These authors reported that the resistance decreased antibiotic sensitivity to ampicillin, chloramphenicol, erythromycin, and tetracycline. Laursen et al. [238] studied if the exposure to a *Lactobacillus plantarum* pediocin could lead to resistance in *L. monocytogenes*. These authors observed changes in the expression of genes regulated by the LisRK system and the SigB and SigL sigma factors; thus, a single exposure to a sublethal concentration of the bacteriocin initiates a response leading to resistance. Some authors have suggested the use of bacteriocin mixtures to overcome the problem of resistance [239,240] although this method is effective only when different mechanisms of resistance are implicated [189]. Cross-resistance among bacteriocins have been observed [237,241,242,243,244] complicating the situation. The stability of *Listeria monocytogenes* mutant’s resistance to LAB bacteriocins (mesenterocin, curvaticin, and plantaricin) was estimated by Rekhif et al. [241] who found that it was maintained for several generations even when the bacteriocins were not present. Furthermore, the mutants resistant to one of the bacteriocins showed a cross-resistance to the two other bacteriocins, but not to nisin. Nisin-resistant variants of *Listeria monocytogenes* as well as resistant to pediocin produced by *Pediococcus pentosaceus* 34, and enterocin produced by *Enterococcus faecium* FH99 were developed by Kaur et al. [242,243]. Cross-resistance between pediocin 34 and enterocin FH 99 was found, but not with nisin. The understanding of bacteriocin resistance is incomplete and further investigations are needed.

### 4.3. Bacteriophages

Bacteriophages are viruses whose only hosts are bacteria [245,246,247]. The addition of virulence factors to the host has been reported, although reductions in virulence have been also described [248] with potential consequences in phage therapy.

Bacteriophages are consumed with the diet because they are natural components of food microbiota [189,249]. The use of bacteriophages to control foodborne bacteria is characterized by the low numbers of non-growing pathogens together with large populations of indigenous bacteria [250,251,252,253,254,255]. Moreover, the repeated use of bacteriophages in food could create resistance [189,256]. The complexity of this issue caused Montville and Chikindas [189] to conclude that “like bacteriocins, bacteriophages are not silver bullets but need to be used from a perspective that considers the microbial ecology of the food”.

## 5. Natural Antimicrobials from Algae and Mushrooms

Macroalgae (seaweeds) and microalgae (diatoms) produce substances with antimicrobial activity. Pharmaceutical and food industries search for promising marine algae derivatives [257,258,259]. There is limited research to evaluate the antimicrobial activity of algae in food biopreservation.

Several authors have studied the antimicrobial characteristics of algae. Herrero et al. [260] carried out the screening for this type of compounds in a macro- and a micro-algae (*Himanthalia elongata* and *Synechocystis* spp., respectively); extracts from both had antioxidant capacities and antimicrobial action against *S. aureus* and *E. coli*. Similar results were obtained by Devi et al. [261] when they studied extracts from *Haligra* spp. active against *S. aureus*. *Hymanthalia elongata*, *Saccharina latissima*, *Laminaria digitate*, *Padina*, and *Dictyota* were reported to have antimicrobial activity against *L. monocytogenes*, *Salmonella*, *Enterococcus faecalis*, *P. aeruginosa*, *B. cereus*, and *E. coli* [262,263].

Algae-derived compounds such as carrageenan and alginates are useful for food coatings and films; these compounds together with other natural antimicrobials will enhance their applications [264]. Carrageenan and alginates have been used in a variety of ways in food industry: forming nanocomposite films enriched with essential oils enhancing its effectiveness against *L. monocytogenes* [265], combined with chitosan and isothiocyanate in a film used for food packaging and active against *C. jejuni* [266], or combined with EDTA in a film increasing the potential to reduce *Salmonella* populations [267]. Alboofetileh et al. [265] developed nanocomposite films with antimicrobial characteristics to control the growth of foodborne pathogens. Firstly, they tested the antibacterial effects of the essential oils of caraway, cinnamon, clove, coriander, cumin, and marjoram against *E. coli*, *S. aureus*, and *L. monocytogenes*. Then, the essential oils of marjoram, clove, and cinnamon—the most potent against the microorganisms—were incorporated into nanocomposite films made with alginate or clay and tested for 12 d. Marjoram (1.5%) showed the highest activity against microorganisms in all matrices decreasing the populations of the three microorganisms up to 6.3, 4.5, and 5.8 log, respectively. Four *Campylobacter jejuni* strains were tested with allyl isothiocyanate contained into an edible coating (with 0.2% κ-carrageenan and 2% chitosan) on vacuum-packaged chicken breasts at 4 °C [266]; the coatings with 50 or 100 μL/g of allyl isothiocyanate reduced the number of cells of the microorganism to levels below the detection limit after 5 d. Olaimat and Holley [267] tested the same carrageenan/chitosan coating previously described for its ability to inhibit the population of *Salmonella* on fresh chicken breasts; the edible coating contained allyl isothiocyanate, mustard, EDTA or their combinations. Coatings containing 50 μL/g of allyl isothiocyanate or 250 mg/g of mustard reduced Salmonella’s counts about 2.3 log CFU/g at 4 °C after 21 d; LAB was also reduced.

Among fungi, mushrooms have antimicrobial and antioxidant capacities [2]. Wild *Laetiporus sulphureus* (Bull.) Murrill fruiting bodies extracts have shown antimicrobial activity in vitro against bacteria such as *Candida albicans*, *Candida parapsilopsis*, *E. coli*, *S. aureus*, *Enterococcus faecalis*, and *S. epidermidis*. Antimicrobial activity was also detected by edible mushrooms extracts of *Aphyllophorales* [268], *Agaricus* [269], and *Armillaria mellea*, *Meripilus giganteus*, *Morchella costata* and *M. elata*, *M. esculenta* var. vulgaris, *M. hortensis*, *M. rotunda*, *Paxillus involutus*, and *Pleurotus eryngii* and *P. ostreatus* [270]. Methanolic extracts of 6 wild edible mushrooms (*Cantharellus cibarius*, *Clavaria vermiculris*, *Lycoperdon perlatum*, *Marasmius oreades*, *Pleurotus pulmonarius*, and *Ramaria formosa*) were used by Ramesh and Pattar [268]. All the isolates showed high content of phenols and flavonoids with antimicrobial activity against several of pathogenic bacteria (*E. coli*, *B. subtilis*, *P. aeruginosa*, and *S. aureus*) and fungi (*Candida albicans*) indicating that the concentrations of the components directly influence the capability of the isolated mushrooms against the microorganisms. Öztürk et al. [269] investigated the fatty acids from *Agaricus essettei*, *A. bitorquis* and *A. bisporus* extracts founding that linoleic and palmitic acids were dominant and active against Gram-positive bacteria (*Micrococcus luteus*, *Micrococcus flavus*, *Bacillus subtilis*, and *Bacillus cereus*). Kalyoncu et al. [270] determined the antimicrobial activities of ethanol extracts from the mushrooms cited above against 11 microorganisms (*Bacillus cereus* and *Bacillus subtilis*, *E. coli*, *Enterobacter aerogenes* and *E. cloacae*, *Enterococcus faecalis*, *Proteus vulgaris*, *S. typhimurium*, *Sarcina lutea*, *S. aureus*, and the yeast *Candida albicans*). *P. ostreatus* and *M. giganteus* were the species with the greater activity against both bacteria and yeast.

Mushrooms’ antimicrobials have not been sufficiently investigated to date for their food application. A fine review of them can be found in Gyawali and Ibrahim [2]. Further information can be found in References [271,272,273,274].

## 6. Future Perspectives

The food industry is receiving increasing pressure from consumers for the use of natural components in its products. The major objective is to use them as natural antimicrobials, and three methods are the most promising in food systems.

### 6.1. Direct Application on Food

The use of natural antimicrobials in food as biopreservatives is often limited due to the smell and taste given to the foods and the difficulties for achieving a good solubility in them [2,275]. Antimicrobial activity against *B. cereus* in rice has been demonstrated after the inclusion of basil, thyme, or oregano essential oils [43,276,277]; 1% of fresh garlic was active against *E. coli* O157 and *S. enterica* serovar Enteritidis in mayonnaise [37]; inhibitory activity against *L. monocytogenes* was found with ground cinnamon in pasteurized apple juice [278] and with essential oils of cinnamon, bark, and clove in semi-skimmed milk [279]; the essential oils of clove, cinnamon, thyme, and bay were active against *L. monocytogenes* in cheese [280,281]; thyme, oregano and lemongrass essential oils combined with modified atmosphere packaging were used to evaluate the inhibition of the total mesophilic population in cabbage and radish sprouts resulting in almost total inhibition of the microorganisms [282].

The shelf-life of meat and meat products has been improved using extracts or essential oils of natural antimicrobials compounds. Thyme and oregano essential oils at 0.1–0.3% were active against meat-based products dipped in them and combined with modified atmosphere packaging [283]. Thyme essential oil combined with nisin significantly decreased the population of *L. monocytogenes* [284] and *E. coli* O157:H7 [285] in minced beef meat under refrigeration conditions. *C. jejuni* populations were reduced in chicken meat after the application of rosemary extracts combined with a pre-freezing period [286] or after the use of *Inula graveolens*, *Laurus nobilis*, *Satureja montana*, and *Pistacia lentiscus* essential oils combined with packaging under microaerophilic conditions [287].

### 6.2. Edible Films and Coatings for Packaging

Food-packaging related industries are showing interest in the use of natural antimicrobials in edible films and coatings to improve food quality. At the same time, consumers’ concerns created by plastic packaging are reduced. Lysozyme-chitosan composite films (a 2% chitosan film with an incorporated solution of 10% lysozyme at 0, 20, 60, or 100%) were developed by Park et al. [150] for improving the antibacterial capacities of chitosan films. The efficacy of chitosan films was enhanced with 60% lysozyme against both *Streptococcus faecalis* (reduction of 3.8 log cycles) and *Escherichia coli* (reductions of 2.7 log cycles). Bayarri et al. [153] determined the properties of lysozyme with methoxyl pectin for developing an edible film with antimicrobial activity. The formation of these complexes considerably decrease the lysozyme antimicrobial activity; however, after their use to manufacture an edible antimicrobial film, the lysozyme release was controlled and the enhancement of the lysozyme release was reported, allowing the use of the edible film to protect foods against microorganisms sensible to lysozyme activity. In their study, Güçbilmez et al. [154] incorporated lysozyme into zein films together with chickpea albumin, bovine serum albumin, and EDTA; that combination gave zein films effective activity against *E. coli* and *Bacillus subtilis*. Chitosan was combined with sodium caseinate to create films by Moreira et al. [162]; these authors evaluated their effectiveness against microbiota of cheese, salami, and carrots. Assays with the film-forming solutions applied on foods showed a significant antimicrobial action on the mesophilic, psychrotrophic, and yeasts and moulds populations with reductions of about 2.0–4.5 log CFU/g.

Edible films containing different extracts or essentials oils have shown their efficacy against foodborne pathogens such as *E. coli* O157:H7 [288,289,290], *L. monocytogenes*, and *Salmonella* [289,290]. Jang et al. [288] manufactured an edible film for strawberries containing 10% of grapefruit seed extract. The film inhibited the growth of *E. coli* O157:H7 and *L. monocytogenes*, and the populations of aerobic bacteria, yeast and moulds decreased after 14 d of storage. Ready-to-eat minimally processed salads were packaged under modified atmosphere conditions with films of polypropylene plus ethylene-vinyl alcohol copolymer (with a 29% ethylene molar content) containing oregano and citral [289,290]. The results showed that antimicrobial activity reduced spoilage microbiota on the salad as well as inhibit the growth of *E. coli*, *S. enterica*, and *L. monocytogenes*; the inhibition was greater against Gram-negative bacteria. Chitosan-based films have been very effective in increasing the shelf-life of different products such as fruits and vegetables [291,292,293,294,295,296], and different meats and products [297,298,299,300]. Cé et al. [291] reported the increase in activity against several bacteria in minimally processed pear after the addition of nisin and peptide P34 to chitosan films: *E. coli*, *B. cereus*, *Clostridium perfringens*, *Lactobacillus acidophilus*, *L. monocytogenes*, *S. aureus*, *S. enteritidis*, *Aspergillus phoenicis*, and *Penicillium stoloniferum*. Films containing natamycin showed similar inhibition than those with chitosan alone. Vodnar et al. [297] developed chitosan-based films with bioactive compounds from green and black teas for the control of *L. monocytogenes* on vacuum-packaged ham steak at 20 °C for 10 d and 4 °C for 8 weeks. *L. monocytogenes* growth was inhibited in a dose-dependent manner: 4% of green tea extract was the most effective at both temperatures; 2% of green tea or 2% and 4% of black tea showed less antibacterial activity. Chitosan lactate was included into low-density polyethylene [298]; these films were applied on the surfaces of red meat and tested against *E. coli*, *L. monocytogenes*, and *S. enteritidis*. The microorganisms on the meat surface were not inhibited; however, a significant extension of the red colour shelf-life was observed. Soy protein edible films with EDTA or nisin have been studied for their physical and antimicrobial properties [301]; the films incorporated with 1% of grape seed extract, 10,000 IU/g of nisin, and 0.16% of EDTA showed the greatest activity against *L. monocytogenes* reducing its population by approximately 3 log CFU/mL. *E. coli* O157:H7 and *S. typhimurium* counts were reduced by approximately 2 and 1 log CFU/mL, respectively.

### 6.3. Nanoparticles and Nanovesicles

Nanotechnology is increasing its role in the food industry and some studies have been carried out over the last years. Applications of nanotechnology to deliver natural antimicrobial compounds in foods are very limited because of the complexity of the technology needed and the food matrix. Eby et al. [149] reported that hen egg-white lysozyme catalyzed the formation of silver nanoparticles that were able to maintain the hydrolase function of the enzyme; they were effective against *E. coli*, *Bacillus anthracis*, *S. aureus*, and *Candida albicans*. These nanoparticles had strong activity against silver-resistant strains of *Proteus mirabilis* as well as against an antibiotic- and silver-resistant *E. coli* strain. Human epidermal keratinocytes studies showed that these nanoparticles were non-toxic at the concentrations used to inhibit microbial growth. Nisin nanoparticles have been tested against *L. monocytogenes* and *S. aureus* with good results [302,303,304], as well as bacteriocin nanovesicles [210] or nanoparticles [208,209] against different pathogens. Zou et al. [302] evaluated the prolonged antimicrobial stability of liposome nanoparticles loaded with nisin against *L. monocytogenes* and *S. aureus*. The MIC of the nanoparticles against both microorganisms was 320 UI/mL, reducing their populations by more than 6 log CFU/mL after 48 and 72 h of incubation, respectively. Field et al. [303] identified a nisin A variant with a serine to glycine change at position 29 and with enhanced efficacy against *S. aureus*. Three more derivatives were developed and tested against *E. coli*, *Cronobacter sakazakii*, and *S. enterica* serovar Typhimurium showing enhanced antimicrobial activity. Encapsulation provides stability to bacteriocins; thus, de Mello et al. [210] encapsulated the peptide pediocin in nanovesicles of soybean phosphatidylcholine. The nanovesicles maintained 50% of the pediocin antimicrobial activity for 13 d at 4 °C against *Listeria monocytogenes*, *L. innocua*, and *L. ivanovii*. Gold nanoparticles with *Lactobacillus acidophilus* CH1 bacteriocin were used by Mossallam et al. [208] against intestinal microsporidiosis in immunosuppressed mice. The anti-microsporidia activity of the bacteriocin was potentiated, showing a sustained reduction in faecal spore shedding and intestinal spore load.

## 7. Conclusions

Since consumers increasingly demand food free of synthetic preservatives, it is necessary to identify and study new alternatives. These new approaches should be useful for controlling foodborne pathogens and for extending the foods’ shelf-life. From the economic point of view, the search for natural antimicrobials must be cost-effective, and one alternative approach would be the mixture of several natural antimicrobials combined with food preservation techniques.

Due to the complexity of food matrices, natural antimicrobial compounds could bind with some food components limiting their action. Nanoparticles and/or nanovesicles have enormous potential in food safety as an effective antimicrobial delivery system, although this technology has raised concerns over consumers’ safety. Therefore, further research is needed to determine the best antimicrobial delivery technology and the best concentrations of such natural antimicrobial compounds.
